# Modeling the impact of thoracic pressure on intracranial pressure

**DOI:** 10.1038/s41526-024-00385-5

**Published:** 2024-04-10

**Authors:** Drayton W. Munster, Beth E. Lewandowski, Emily S. Nelson, R. K. Prabhu, Jerry G. Myers Jr

**Affiliations:** 1 NASA Glenn Research Center, 21000 Brookpark Road, Cleveland, OH 44135 USA; 2 Universities Space Research Association, 21000 Brookpark Road, Cleveland, OH 44135 USA

**Keywords:** Risk factors, Pathogenesis

## Abstract

A potential contribution to the progression of Spaceflight Associated Neuro-ocular Syndrome is the thoracic-to-spinal dural sac transmural pressure relationship. In this study, we utilize a lumped-parameter computational model of human cerebrospinal fluid (CSF) systems to investigate mechanisms of CSF redistribution. We present two analyses to illustrate potential mechanisms for CSF pressure alterations similar to those observed in microgravity conditions. Our numerical evidence suggests that the compliant relationship between thoracic and CSF compartments is insufficient to solely explain the observed decrease in CSF pressure with respect to the supine position. Our analyses suggest that the interaction between thoracic pressure and the cardiovascular system, particularly the central veins, has greater influence on CSF pressure. These results indicate that future studies should focus on the holistic system, with the impact of cardiovascular changes to the CSF pressure emphasized over the sequestration of fluid in the spine.

## Introduction

Unique to spaceflight, Spaceflight Associated Neuro-ocular Syndrome (SANS) manifests as a reduction in an astronaut’s visual acuity and other anatomical changes suggestive of early-stage ocular disorders, such as optic disc edema, cotton wool spots, and choroidal folds^1,2^. The etiology associated with the manifestation of SANS in some astronauts and not others remains unclear but is likely a multifactorial response to the microgravity and vehicle environment^3,4^. Astronauts experience several well-documented physiological changes when exposed to the microgravity environment, one of the most prominent being the cephalad, or headward, redistribution of fluid^5^. Vascular deconditioning appears following these cephalad fluid redistribution changes, resulting in an incomplete orthostatic response on return to a terrestrial environment with standard gravity (1-G)^6–9^. NASA and Russian Space agencies utilize a number of inflight countermeasures or pre-return-to-earth procedures, such as fluid loading and exercise^10^, compression garments^10,11^, and lower body negative pressure (LBNP)^12^ to mitigate the likelihood of orthostatic intolerance.

The prevalent hypothesis relates the fluid shift contribution to SANS symptoms to either elevated cerebrospinal fluid (CSF) pressure or fluid compartmentalization in the region of the eye during microgravity^1^. Recent studies illustrate the potential for moderately elevated intracranial pressure (ICP) in microgravity to pressure levels between 1-G supine and standing orientations. Unlike the terrestrial environment in which CSF pressure varies due to postural changes, the acute change fluid redistribution in microgravity produces mildly and chronically elevated CSF pressure^13^. From this, it is has been hypothesized that SANS symptoms manifest less from vascular changes and more from the relation of CSF redistribution due to physiological pressure changes, such as reduction in thoracic pressure or changes in CSF and cranial blood volume^14,15^. Specifically, thoracic pressure change in microgravity is postulated to mediate the mechanisms of fluid redistribution in the spinal dural space in a manner similar to mediation seen during standing^16^. Although the volume of the CSF in the spinal cord subarachnoid space, encapsulated by the spinal dural sac is less than 20% of the total CSF volume^17^, the contribution of these CSF redistributions resulting from microgravity-associated physiological changes are currently unresolved.

In the context of this manuscript, we will refer to thoracic pressure as associated with the average pressure in the entire thoracic cavity, including the pleural, pulmonary, blood, interstitial, and air domains. Defined in this manner, direct measurement of the averaged thoracic pressure historically relies on inferential measures, such as esophageal pressure^18^ or thoracic impedance^19^. Parabolic flight testing shows that the reduction in esophageal pressure during microgravity portions of the flight exceeds 5 mHg on average, compared to <2 mmHg average reduction in central venous pressure (CVP)^20^. Under these conditions, thoracic pressure reduction is related to both the acute cephalad fluid shift^20^ and the removal of so-called tissue compressive forces^21^, typically described as the pressure generated by the weight of the tissue surrounding fluid holding vessels. However, mean arterial pressure (MAP) may also exhibit a steady drop during most of the microgravity portion of parabolic flight, reaching as much as 30 mmHg below its maximum value at the initiation of the microgravity period^22^. This is likely because of hydrostatically affected regulation factors due to cephalad fluid shift. Adding to these observations, parabolic flight studies demonstrate that the average pressure of the intra-jugular vein increases during the microgravity portion of the parabolic flight^23^ and the flow may stagnate or reverse during the cardiac cycle in more prolonged microgravity settings^24^. This illustrates the complex interaction of fluid redistribution and other gravity-influenced mechanisms derived from parabolic flight studies and that the time-course measurements have not always reached homeostatic pressure balance in the <20 s microgravity period, further complicating interpretation of these findings for long-duration missions.

Lumped-parameter computational models of the cardiovascular system represent an established tool for investigating the impact of cephalad fluid shift and thoracic pressure in altered gravity conditions^25–28^. Early models, premised on the work of Guyton^29^, provide an understanding of the importance of the decrease in hydrostatically induced thoracic pressure, and thus a change in intrapleural (extracardiac) pressure, in explaining the paradoxical relation of stroke volume and cardiac output through microgravity-induced compounding of physiological responses^25^. Models that include increasingly complex representations of the cardiovascular blood compartment interactions and regulatory mechanisms relate vascular deconditioning to the onset of syncope and orthostatic intolerance^26,28,30^ and the relative contribution of altered (artificial) gravity to preventing orthostatic deconditioning^31^. Fewer lumped-parameter models include intracranial blood and CSF compartments^32–35^. Those who implement such tools often simplify assumptions, in particular, Monro-Kellie conditions, where a constraint restricts the net fluid volume in the head to a fixed quantity due to the presence of the rigid cranium.^32,33,35^ or CSF and blood compartment interactions^21^. Regardless of these limitations, reduced gravity and tilt simulations with lumped parameter models that include the intracranial blood, with or without CSF interactions, predict a moderate reduction in ICP between 1 mmHg^35^ and 4 mmHg^21^ under microgravity conditions with respect to the supine position. Although these findings qualitatively match invasive CSF pressure measurements under acute microgravity conditions (~2–4 mmHg)^13^, they do not include the potential regulation of the CSF pressure via repositioning of fluid to the spinal dural sac via changes in simulated thoracic pressure which may be important at longer durations of microgravity exposure.

In this study, we investigate the thoracic-to-spinal dural sac transmural pressure contribution to CSF regulation proposed by Laurie, et al.^15^ through a set of numerical investigations simulating microgravity thoracic pressure change and the role such changes play on CSF pressure regulation. The numerical study extends a published lumped parameter model of CSF and blood interactions in the cranial vault^33^ to include Monro-Kellie volume constraints, the existence of multiple cranial drainage pathways, and dynamic venous compliance contributions. We demonstrate the model’s credibility in CSF pressure prediction by comparative validation to analogous tilt table studies. To elicit an understanding of the magnitude and pathways by which microgravity thoracic pressure changes contribute to changes in CSF pressure, we present the results of two parametric simulations that seek to isolate specific influences on CSF spaces. Each study complements the other, and together they inform a more comprehensive picture of the role of thoracic pressure in CSF pressure regulation.

## Methods

### The head model

The present mathematical model (henceforth, head model) is an extension of the lumped-parameter model introduced by Stevens, et al.^33^, to study steady-state solutions to CSF Infusion, Head-Down Tilt (HDT), and Microgravity challenges. The purpose of the model described herein is to investigate changes in fluid drainage from the head and changes in the pressures and volumes of cranial compartments in response to prescribed changes in thoracic pressure, MAP, and CVP. Except where otherwise noted, we shall use the pressure of Ventricular CSF compartment and ICP interchangably. Since no human studies were performed and all parameter data is available via published sources, ethical approval was not sought for this work.

**Fig. 1:**
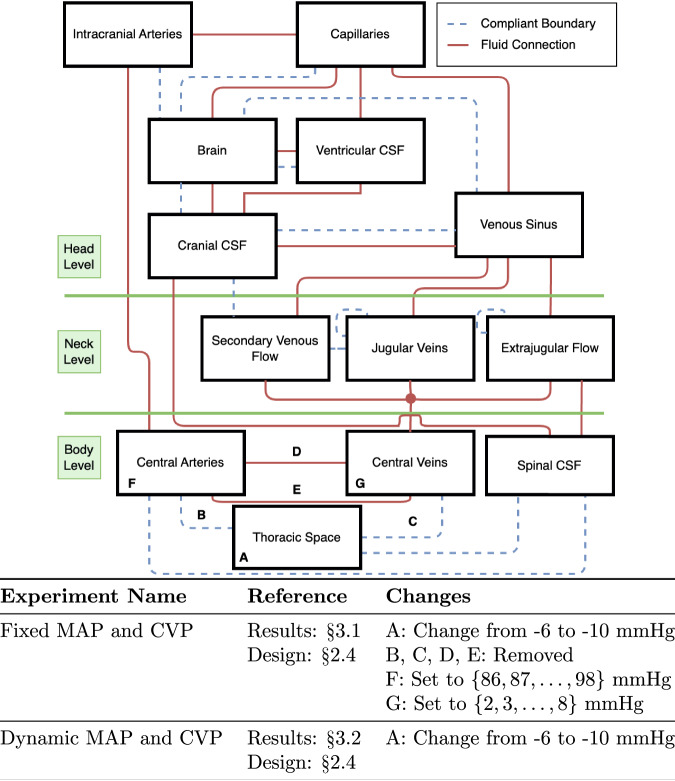
Connectivity Diagram of the Head Model with the Changes for the Numerical Experiments.

Briefly, the head model, illustrated in Fig. 1, abstracts the fluid holding anatomy of the cranial space as a series of discrete, compliant compartments with fixed, resistive flow paths specifying the movement of blood and CSF through the cranial volume. Imposing a conservation of mass constraint at each compartment allows for the simultaneous solution of the flow and volume distribution within each fluid space.

Notable changes from the original Stevens model include partitioning the “Venous Sinus/Jugular Vein” compartment into a series of three “neck-level” compartments and a cranial compartment. Additionally, the “Extraventricular CSF” compartment is partitioned into two new compartments. The new neck and CSF compartments are as follows:

1. Cranial CSF: cranial compartment capturing the volume of CSF present in cerebral cisterns and cranial subarachnoid space;
2. Spinal CSF: “body-level” compartment capturing the volume of CSF present in spinal subarachnoid spaces;
3. Venous Sinus: cranial compartment capturing the blood flow and volume of the venous sinus which communicates with the Cranial CSF and the “neck-level” compartments;
4. Jugular Veins: “neck-level” compartment capturing flow through the left and right internal jugular veins;
5. Secondary Veins: “neck-level” compartment capturing the flow through the vertebral veins and other (smaller) spinal venous structures;
6. Extrajugular: “neck-level” compartment capturing the flow through interstitial spaces, deep neck veins, and any other extra-spinal drainage.

This division of “neck-level” compartments is inspired by the ultrasound analysis completed in refs. 36 and 37, MRI reconstructions and assessments given in ref. 38, and descriptions given in ref. 39. The division of cerebral drainage between the three “neck-level” compartments (specified as a percentage of total drainage) was taken from the mean values for healthy control subjects in ref. 38 which are compatible with those given in refs. 36,37.

In addition to the conservation of mass, we enforce two additional constraints:

1. Pressure in the Thoracic Compartment: the pressure for the Thoracic compartment is specified as a function of time.
2. Monro-Kellie constraint: Originally considered by Monro and, later, Kellie^40^, the volume of the cranial compartment is fixed. The Monro-Kellie constraint encompasses all compartments labeled as at the “Head Level” in Fig. 1.

While the first of these simply replaces the relevant dynamic equation, the second represents an additional algebraic constraint imposed upon the system. Therefore, the system of ordinary differential equations is over-determined. To resolve this, we use techniques from geometric numerical integration^41,42^ to project the equations onto a lower-dimensional manifold where the constraints are satisfied. This is equivalent to eliminating equations/variables by solving the algebraic constraints for a particular variable, but in a more general setting. This will be discussed in more detail in “Model construction”.

### Model construction

To derive the head model, much like in similar lumped-parameter models^32,33,43^, we make the following assumptions:

- All fluids (i.e., blood and CSF) are incompressible and isothermal.
- Flow across the blood-brain barrier (i.e. between the Capillaries and Brain compartments) is given by the Starling-Landis Equation:
  $${Q}_{{{{\rm{Capillaries}}}},{{{\rm{Brain}}}}}={K}_{{{{\rm{CB}}}}}\left[({P}_{{{{\rm{Capillaries}}}}}-{P}_{{{{\rm{Brain}}}}})-{\sigma }_{{{{\rm{CB}}}}}({\pi }_{{{{\rm{C}}}}}-{\pi }_{{{{\rm{B}}}}})\right],$$
  where *Q*_Capillaries,Brain_ is the flow (mL min^−1^) from the Capillaries compartment to the Brain compartment, *K*_CB_ is the filtration coefficient (mL min^−1^ mmHg^−1^), *P*_Capillaries_ is the pressure (mmHg) in the Capillaries compartment, *P*_Brain_ is the pressure (mmHg) in the Brain compartment, *σ*_CB_ is the reflection coefficient, *π*_C_ is the blood colloid osmotic pressure (mmHg), and *π*_B_ is the brain interstitial fluid osmotic pressure (mmHg). The values for these parameters can be found in Table 1. The only osmotic forces considered are those due to differences in protein concentration.

**Table 1:** Parameter Values

Parameter	Description	Value (unit)	Source
*K*_*CB*_	Filtration coefficient for the Starling-Landis equation	0.066 (mL min^−1^ mmHg^−1^)	^33^
*σ*_*CB*_	Reflection coefficient for the Starling-Landis equation	1 (unitless)	^33^
*π*_*C*_	Blood colloid osmotic pressure	21.5 (mmHg)	^33^
*π*_*B*_	Interstitial fluid osmotic pressure	0 (mmHg)	^33^
*N*	Ratio of asymptotic to peak compliance	0.01 (unitless)	^26^
*α*_Neck_	Compliance steepness factor for neck-level compartments	0.40026 (mmHg^−1^)	^26^
*α*_Central veins_	Compliance steepness factor for the Central Veins Compartment	0.29352 (mmHg^−1^)	^26^
*Z*_Heart_	Fluidity from the Central Veins to the virtual heart compartment	1250 (mL min^−1^ mmHg^−1^)	^26^
*S*	Heart pumping efficiency	1 (unitless)	^26^
*HR*_0_	Baseline heartrate	61.6 (beats min^−1^)	^26^
*SV*	Baseline stroke volume	77.2 (mL beat^−1^)	^26^
*α*_Heart_	Cardiac output coefficient	7.809 (unitless)	^26^
*β*	Cardiac output steepness coefficient	0.381 (mmHg^−1^)	^26^
*τ*	Characteristic time for heartrate	4 (seconds)	^49^
*α*_*σ*_	Coefficient for sympathetic activation	1.15 (unitless)	^49^
*β*_*σ*_	Coefficient for parasympathetic activation	0.34 (unitless)	^49^
*γ*_*σ*_	Coefficient for baseline activation	0.595 (unitless)	^49^
*ν*	Slope coefficient for sympathetic and parasympathetic activation	7 (unitless)	^49^
- When not otherwise specified, all other flows are proportional to the pressure differential between compartments. That is,
  $${Q}_{ij}={Z}_{ij}({P}_{i}-{P}_{j}),$$
  where *Q*_*ij*_ denotes the flow from compartment *i* to compartment *j* in mL min^−1^, *Z*_*ij*_ is the fluidity (or inverse of flow resistance) in mL min^−1^ mmHg^−1^, and *P*_*i*_, *P*_*j*_ refer to the pressures in compartments *i* and *j*, respectively, in mmHg.
- The change in volume between two compartments that share a compliant boundary is linear in the change in the pressure differential between them. That is,
  $$\frac{{{{\rm{d}}}}{V}_{ij}}{{{{\rm{d}}}}t}={C}_{ij}\frac{{{{\rm{d}}}}}{{{{\rm{d}}}}t}\left({P}_{i}-{P}_{j}\right),$$
  where *V*_*ij*_ denotes the volume of the cup formed by the interface between compartments *i*, *j* in mL and *C*_*ij*_ = *C*_*ji*_ is the local compliance between the compartments in mL mmHg^−1^.
- CSF production is constant as a result of sufficiently robust regulatory mechanisms at the pressure levels of interest^33^. (See Table 2.)

**Table 2:** Mean Flows between Compartments at Steady-State

Source Compartment	Destination Compartment	Flow (mL min^−1^)	Source
Central Arteries	Intracranial Arteries	795.	^52^
	Central Veins	4505	^52^
Intracranial Arteries	Capillaries	795.	–
Capillaries	Brain	0.13	^33^
	Ventricular CSF	0.30	^33^
	Venous Sinus	794.57	–
Ventricular CSF	Brain	0	^33^
	Cranial CSF	0.30	–
Brain	Cranial CSF	0.13	–
Cranial CSF	Venous Sinus	0.30	^33^
	Spinal CSF	0.13	^33^
Spinal CSF	Extrajugular	0.13	–
Venous Sinus	Secondary Venous	43.72	^36–38^
	Extrajugular	140.70	^36–38^
	Jugular	610.48	^36–38^
Jugular	Central Veins	610.48	–
Secondary Venous	Central Veins	43.72	–
Extrajugular	Central Veins	140.70	–

Sources labeled “–” indicate conservation of volume at steady-state.

By imposing conservation of mass (equiv. volume, since the fluids are assumed incompressible) in each compartment, we form the following system of differential equations:

1 $${{{\bf{C}}}}(t,{{{\bf{P}}}}(t))\cdot \frac{{{{\rm{d}}}}{{{\bf{P}}}}(t)}{{{{\rm{d}}}}t}={{{\bf{Z}}}}(t,{{{\bf{P}}}}(t))\cdot {{{\bf{P}}}}(t)+{{{\bf{F}}}}(t,{{{\bf{P}}}}(t)),$$

where $${{{\bf{P}}}}(t)=\left(\begin{array}{r}{P}_{1}(t)\\ \vdots \\ {P}_{n}(t)\end{array}\right)$$ is the vector of compartmental pressures,

$${{{\bf{C}}}}(t,{{{\bf{P}}}}(t))=\mathop{\sum }\limits_{i=1}^{n}\mathop{\sum }\limits_{j=i+1}^{n}({{{{\bf{E}}}}}_{i,i}+{{{{\bf{E}}}}}_{j,j}-{{{{\bf{E}}}}}_{i,j}-{{{{\bf{E}}}}}_{j,i}){C}_{ij}(t,{{{\bf{P}}}}(t))+\mathop{\sum }\limits_{i=1}^{n}{{{{\bf{E}}}}}_{i,i}{C}_{ii}(t,{{{\bf{P}}}}(t))$$

is the compliance matrix,

$${{{\bf{Z}}}}(t,{{{\bf{P}}}}(t))=\mathop{\sum }\limits_{i=1}^{n}\mathop{\sum }\limits_{j=1}^{n}({{{{\bf{E}}}}}_{i,j}+{{{{\bf{E}}}}}_{j,i}-{{{{\bf{E}}}}}_{i,i}-{{{{\bf{E}}}}}_{j,j}){Z}_{ij}(t,{{{\bf{P}}}}(t))$$

is the fluidity matrix, **F**(*t*, **P**(*t*)) represents any forced flows per compartment, **E**_*i*,*j*_ = **e**_*i*_ ⊗ **e**_*j*_, and **e**_*i*_ is the *i*th canonical basis vector. To track changes in volume and enforce constraints, we can extend this system:

2 $$\left[\begin{array}{cc}{{{\bf{C}}}}(t,{{{\bf{P}}}}(t))&{{{\bf{0}}}}\\ {{{\bf{0}}}}&{{{\bf{I}}}}\end{array}\right]\cdot \frac{{{{\rm{d}}}}}{{{{\rm{d}}}}t}\left(\begin{array}{l}{{{\bf{P}}}}(t)\\ {{{\bf{V}}}}(t)\end{array}\right)=\left[\begin{array}{cc}{{{\bf{Z}}}}(t,{{{\bf{P}}}}(t))&{{{\bf{0}}}}\\ {{{\bf{Z}}}}(t,{{{\bf{P}}}}(t))&{{{\bf{0}}}}\end{array}\right]\cdot \left(\begin{array}{l}{{{\bf{P}}}}(t)\\ {{{\bf{V}}}}(t)\end{array}\right)+\left(\begin{array}{l}{{{\bf{F}}}}(t,{{{\bf{P}}}}(t))\\ {{{\bf{F}}}}(t,{{{\bf{P}}}}(t))\end{array}\right),$$

where **0** and **I** are suitably sized zero and identity matrices, respectively.

As in^33^, we assign a 0.2 mmHg difference in pressure between the Spinal CSF compartment and the Ventricular CSF compartment to represent the transmantle pressure. In order for CSF to flow in the stead-state condition from the Ventricular CSF compartment, through the Cranial CSF compartment, to the Spinal CSF compartment, it is necessary for the Cranial CSF compartment to take a baseline pressure between 11.0 and 11.2 mmHg. Our numerical experimentation indicates that the model is not sensitive to the value within this range, so a mean value of 11.1 mmHg is assigned to the baseline Cranial CSF compartment pressure.

Also as in^33^, we use the approximation that the fluidity between the Brain and Ventricular CSF compartments is 1000 times the fluidity across the blood-brain barrier, i.e., between the Capillaries and Brain compartments. We therefore set *Z*_Ventricular CSF,Brain_ = 1000*K*_CB_, where *K*_CB_ is the filtration coefficient from the Starling-Landis equation.

We compute the remainder of the fluidities using measured mean pressures and flows between compartments. That is, we compute

3 $${Z}_{ij}=\frac{{\bar{Q}}_{ij}}{{\bar{P}}_{i}-{\bar{P}}_{j}},$$

where ${\bar{Q}}_{ij}$ is the mean flow rate from compartment *i* to compartment *j*, and ${\bar{P}}_{i},{\bar{P}}_{j}$ are the mean pressures for those compartments. This ensures that the given mean pressures are a steady-state solution of the differential equation. The values and their sources used for this paper are given in Tables 2 and 3.

**Table 3:** Baseline Compartment Pressures at Steady-State

Compartment Name	Pressure (mmHg)	Source
Central Arteries	92.	^33^
Intracranial Arteries	82.	^33^
Capillaries	34.64	^33^, Eq. (12)
Brain	11.2	^33^
Ventricular CSF	11.2	^33^, Eq. (11)
Cranial CSF	11.1	§2.2
Venous Sinus	6.6	^39^
Jugular	5.683	^53^
Secondary Venous	5.80	^53^
Extrajugular	5.80	^53^
Spinal CSF	11.	^33^
Central Veins	5.	^53^
Thoracic	-6.	^32^

The baseline compliance values at mean pressures are given in Table 4. We use the pressure-dependent cranial compliance functions from^32,43,44^ for the compliances between the following compartments:

- Brain and Ventricular CSF
- Brain and Venous Sinus
- Brain and Cranial CSF
- Venous Sinus and Cranial CSF.

Details and derivation may be found in the original sources, we present the approach in brief below. The general form is given by

4 $${C}_{{{{\rm{FV}}}}}({P}_{i},{P}_{j})={C}_{0}\exp (-r| {P}_{i}-{P}_{j}{| }^{\gamma }),$$

where we use the parameter values *C*_0_ = 6.5333, *r* = 0.633431, and *γ* = 0.604229^32,33,43^. As in^32^, we portion the compliance values by relative volumes and assign the bulk (95%) of compliance values to interfaces with the venous compartments. This yields the following pressure-dependent compliance formulae:

5 $${C}_{{{{\rm{Brain}}}},{{{\rm{Ventricular}}}}\,{{{\rm{CSF}}}}}=0.05\cdot \left(\frac{23}{140}\right)\cdot {C}_{{{{\rm{FV}}}}}\left({P}_{{{{\rm{Brain}}}}},{P}_{{{{\rm{Ventricular}}}}\,{{{\rm{CSF}}}}}\right),$$

6 $${C}_{{{{\rm{Brain}}}},{{{\rm{Venous}}}}\,{{{\rm{Sinus}}}}}={C}_{{{{\rm{FV}}}}}\left({P}_{{{{\rm{Brain}}}}},{P}_{{{{\rm{Venous}}}}\,{{{\rm{Sinus}}}}}\right),$$

7 $${C}_{{{{\rm{Brain}}}},{{{\rm{Cranial}}}}\,{{{\rm{CSF}}}}}=0.05\cdot \left(\frac{87}{140}\right)\cdot {C}_{{{{\rm{FV}}}}}\left({P}_{{{{\rm{Brain}}}}},{P}_{{{{\rm{Cranial}}}}\,{{{\rm{CSF}}}}}\right),$$

8 $${C}_{{{{\rm{Venous}}}}\,{{{\rm{Sinus}}}},{{{\rm{Cranial}}}}\,{{{\rm{CSF}}}}}=0.95\cdot \left(\frac{87}{140}\right)\cdot {C}_{{{{\rm{FV}}}}}\left({P}_{{{{\rm{Venous}}}}\,{{{\rm{Sinus}}}}},{P}_{{{{\rm{Cranial}}}}\,{{{\rm{CSF}}}}}\right).$$

For the neck-level (Jugular, Secondary Venous, Extrajugular) and Central Veins compartments, we use the formulation for venous compliance as given by^26^ to compute a total compliance value for the compartment. That is, for compartment *i*, the total compliance is determined by

9 $${C}_{i}(t,{{{\bf{P}}}}(t))={C}_{i}^{0}\left(N+\frac{(1-N)}{\cosh \left({\alpha }_{i}({P}_{i,{{\mbox{trans}}}}(t)-4)\right)}\right),$$

where ${C}_{i}^{0}$ is the peak compliance of the compartment, *N* represents the ratio of asymptotic to peak compliance, *α*_*i*_ is the compliance steepness factor, and *P*_*i*,trans_ is the transmural pressure of the compartment. For the neck-level compartments, the compartment pressure is used for the transmural pressure. For the Central Veins compartment, the transmural pressure is computed as *P*_Central Veins_(*t*) − *P*_Thoracic_(*t*). The peak compliance values, ${C}_{i}^{0}$, are computed by matching the baseline compliance values from Table 4 with Equation (9) using the mean pressures given in Table 3 and parameter values from Table 1.

**Table 4:** Baseline compliance values

Compartment 1	Compartment 2	Compliance (mL mmHg^−1^)	Source
Brain	Intracranial Arteries	0.021	^32^
	Ventricular CSF	0.054	^32,§2.2.3^
	Capillaries	0.69	^32^
	Venous Sinus	1.33	^32,§2.2.3^
Cranial CSF	Brain	0.16	^32,§2.2.3^
	Venous Sinus	0.82	^32,§2.2.3^
	Secondary Venous	0.010	^33^^a^
Spinal CSF	Central Arteries	0.0057	^32^
Jugular	Secondary Venous	0.40	^53^
	Jugular	1.35	^53^
Extrajugular	Extrajugular	16.38	^32,39,52^
Thoracic	Spinal CSF	0.034	^32,54^
	Central Veins	51.86	^32^
	Central Arteries	1.62	^32^

Note: Since *C*_*ij*_ = *C*_*ji*_, the order of the compartments is arbitrary.

^a^ Reassigned from the “Venous Sinus/Jugular Veins” compartment.

To enforce linear constraints such as the Monro-Kellie constraint, we make use of tangent space parameterization techniques from ref. 42. This is equivalent to algebraically eliminating equations using the constraint equations to solve for given variables. By using this parameterization, the constraints will be satisfied to machine precision at every time step. We consider the following general form for a system of ordinary differential equations:

10 $${{{\bf{M}}}}({{{\bf{X}}}}(t))\frac{{{{\rm{d}}}}}{{{{\rm{d}}}}t}\left({{{\bf{X}}}}(t)\right)={{{\bf{G}}}}({{{\bf{X}}}}(t))$$

subject to the linear constraints

11 $${{{\bf{A}}}}{{{\bf{X}}}}(t)-{{{\bf{B}}}}={{{\bf{0}}}}.$$

Let **Q** be an orthonormal basis for the null space of **A**, that is, **Q**^*T*^**Q** = **I** and **Ay** = **0** implies **y** = **Qz** for some **z**. Assuming that **AX**(0) − **B** = **0**, we can write **X**(*t*) = **QZ**(*t*) + **X**(0), where **Z**(0) = **0**. The system in Eq. (10) then can be written as the over-determined system:

12 $${{{\bf{M}}}}({{{\bf{X}}}}(t)){{{\bf{Q}}}}\frac{{{{\rm{d}}}}}{{{{\rm{d}}}}t}\left({{{\bf{Z}}}}(t)\right)={{{\bf{G}}}}({{{\bf{X}}}}(t)).$$

Premultiplication of Eq. (12) with **Q**^*T*^ yields our reduced set of equations:

13 $$\underbrace{\left({\mathbf{Q}}^T {\mathbf{M}}({\mathbf{X}}(t)) {\mathbf{Q}}\right)}_{\widehat{{\mathbf{M}}}({\mathbf{Z}}(t))} \frac{{\mathrm{d}}}{{\mathrm{d}}t}\left( {\mathbf{Z}}(t) \right) = \underbrace{{\mathbf{Q}}^T {\mathbf{G}}({\mathbf{X}}(t))}_{\widehat{\mathbf{G}}({\mathbf{Z}}(t))},$$

which may be solved using standard techniques.

### Model Validation

To validate the construction and calibration of the head model, we turn to clinical data for tilt-table studies, where participants are placed in a supine position and hemodynamic and CSF pressure data is collected while subjected to a series of tilt angles. While not in a supine position (i.e., at tilt angles other than 0), the body is subject to hydrostatic effects which can induce fluid shifts and pressure changes.

**Fig. 2:**
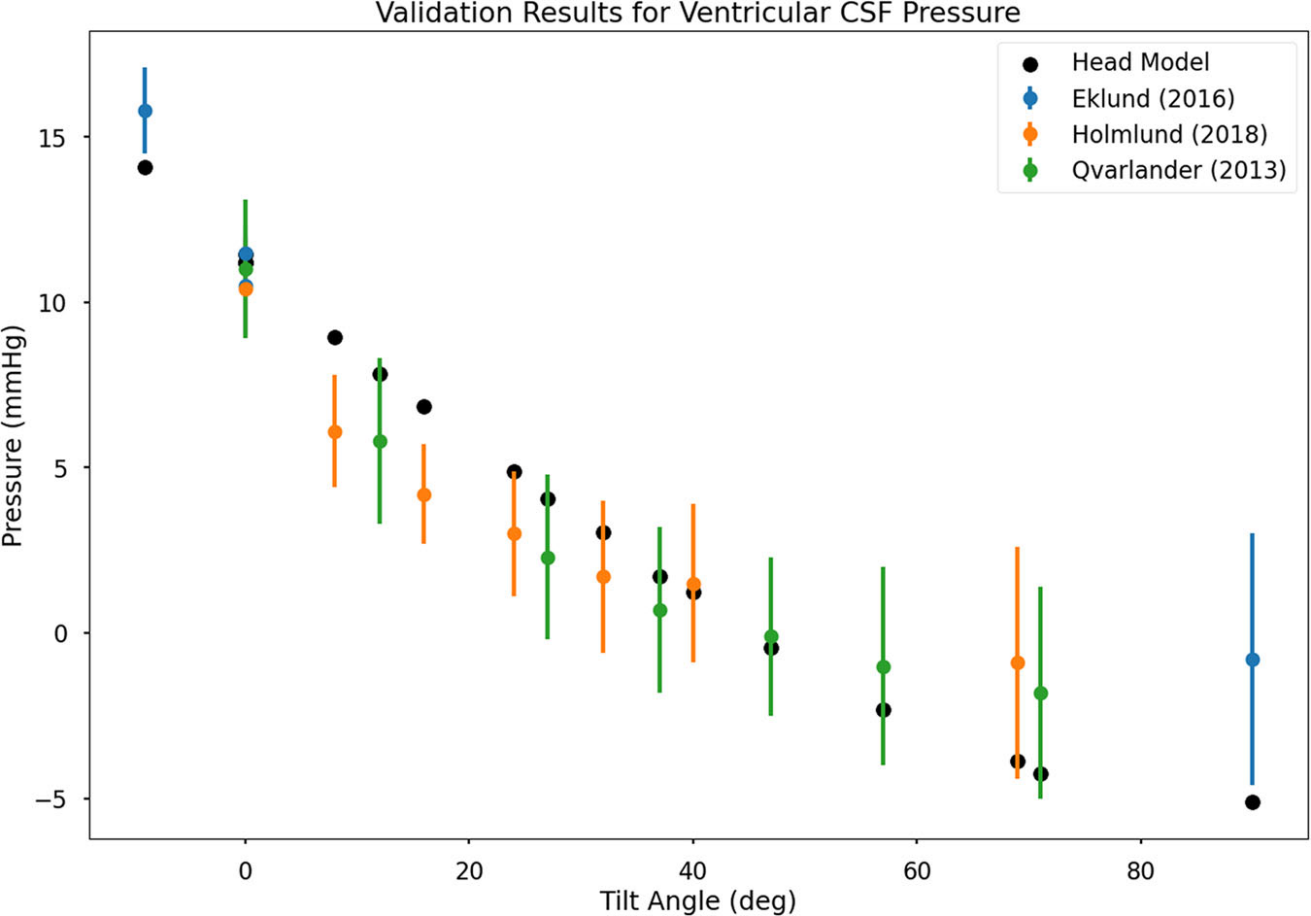
Comparison of model predictions of ventricular CSF compartment pressure against tilt-table studies. Error bars indicate mean ± one standard deviation.

We replicated the experimental conditions of three tilt-table studies that included data on ICP (mapped here to the Ventricular CSF pressure)^45–47^. The results of these validation are presented in Fig. 2. We note a fairly reasonable agreement with the referent studies with a slight under-prediction (in terms of magnitude) for small tilt-angles and a slight over-prediction (in terms of magnitude) for large tilt-angles. Considering the head model does not incorporate a lower body or arteriole-regulatory mechanisms that would be present in response to large-scale fluid shifts such as those imparted by hydrostatics, this level of agreement seems reasonable.

### Experimental configurations

To help orient the reader, we summarize the changes made for each numerical experiment in Fig. 1. Note that the model is held in the supine position for each experiment, removing hydrostatic effects. Each experiment is run until the model has converged to a steady-state.

For the Fixed MAP and CVP study and to isolate the effects of the compliant boundary between the Thoracic and the Spinal CSF compartments, we make the following changes to the head model:

- the pressure in the Thoracic compartment linearly changes from -6 – -10 mmHg over the first minute of the experiment, that is,
  $${P}_{{{{\rm{Thoracic}}}}}(t)=\left\{\begin{array}{ll}-6\quad &t\, <\, 0\\ -4t-6\quad &0\le t\le 1\\ -10\quad &t\, >\, 1\end{array}\right.,$$
- the pressure in the Central Arteries compartment is fixed to a specified value, and
- the pressure in the Central Veins compartment is fixed to a specified value.

In this study, we consider a total of 91 combinations of (fixed) pressure in the Central Arteries and Central Veins compartments. In particular, we consider a Central Arteries pressure from $\left\{86,87,\ldots ,98\right\}$ mmHg (representing ± 6 mmHg from baseline) and a Central Veins pressure from $\left\{2,3,\ldots ,8\right\}$ mmHg (representing ± 3 mmHg from baseline). This range of values covers the baseline Central Arteries and Central Veins compartment pressures used by the models in refs. 32,33 in a symmetric interval and is intended to demonstrate the independence of the results from any particular fixed values for the pressures.

The model is first initialized with the given pressure for the Central Arteries and Central Veins compartments and allowed to run until steady-state. These steady-state pressures are then used as the initial condition for the experiment in which the pressure in the Thoracic compartment is varied. While this linear pressure profile for the Thoracic compartment does not correspond to any particular experimental or observed profile, the analysis was repeated for various non-linear profiles and over a range of times. Changes to the length of time during which the changes take place as well as non-linear profiles do impact the magnitude of the response: shorter time intervals and “sharper” profiles (i.e., those with larger rates of change) will increase the magnitude of the initial “ring” but do not alter the steady-state solution nor the qualitative behavior of the solution. Therefore, a linear profile was used for ease of implementation as a dynamic equation.

For the Dynamic MAP and CVP study and to determine the impact of changes in pressure in the Thoracic compartment on cardiac output, we make the following change to the head model:

- the pressure in the Thoracic compartment linearly changes from -6 to -10 mmHg over the first minute of the experiment, that is,
  $${P}_{{{{\rm{Thoracic}}}}}(t)=\left\{\begin{array}{ll}-6\quad &t\, <\, 0\\ -4t-6\quad &0\le t\le 1\\ -10\quad &t\, >\, 1\end{array}\right.,$$
- the cardiac output is as originally described in ref. 25 and used in ref. 26.

That is, the cardiac output is modeled as a mass-balance between pressure-driven flow from the Central Veins to the heart and a Starling-like flow from the heart to the Central Arteries. At each heartbeat, the pressure for a virtual heart compartment, *P*_Heart_, is determined so that the following equation is satisfied:

14 $${F}_{{{{\rm{in}}}}}({P}_{{{{\rm{Heart}}}}})={F}_{{{{\rm{out}}}}}({P}_{{{{\rm{Heart}}}}}).$$

*F*_in_ represents the pressure-driven flow into the heart and is given by

15 $${F}_{{{{\rm{in}}}}}({P}_{{{{\rm{Heart}}}}})=\max \left({Z}_{{{{\rm{Heart}}}}}\left({P}_{{{{\rm{Central}}}}\,{{{\rm{Veins}}}}}-\max \left({P}_{{{{\rm{Heart}}}}},{P}_{{{{\rm{Collapse}}}}}\right)\right),0\right),$$

where *Z*_Heart_ represents the fluidity between the Central Veins compartment and the virtual heart compartment and *P*_Collapse_ represents the pressure of partial collapse^26^. In our application, the partial collapse pressure is given by *P*_Collapse_ = *P*_Thoracic_ + 2.

*F*_out_ represents the flow from the heart into the Central Arteries compartment and is given by

16 $${F}_{{{{\rm{out}}}}}({P}_{{{{\rm{Heart}}}}})=\frac{S\cdot \,{{\mbox{HR}}}(t)\cdot {{\mbox{SV}}}\,\cdot C}{1+{\alpha }_{{{{\rm{Heart}}}}}\exp \left(-\beta \left({P}_{{{{\rm{Heart}}}}}-{P}_{{{{\rm{External}}}}}\right)\right)},$$

where *S* represents the effectiveness of the heart pumping (values *S* < 1 can be used to model damaged/atrophied tissue), HR(*t*) is the heart rate (beats min^−1^), SV is the stroke volume (mL beat^−1^), and *C* is a subject-specific tuning factor used to fit the cardiac output to the specified baseline flow rate. In our application, the external pressure, *P*_External_ is equal to the pressure in the Thoracic compartment, *P*_Thoracic_. This reflects the impact that the thoracic space pressure has on diastolic filling^48^.

Once a virtual heart pressure that achieves this balance is found, the resulting flow rate is used for the duration of the heartbeat ((HR(*t*))^−1^ minutes).

To model the baroreceptor regulatory mechanism, we use the formulation developed in ref. 49. Since the activation amounts are a time-average of the previous cardiac cycle, we may solve^49^ Eq. 5 to yield

17 $${{{\mbox{HR}}}}^{[n+1]}={\sigma }^{[n]}+\left({{{\mbox{HR}}}}^{[n]}-{\sigma }^{[n]}\right)\exp \left(-\frac{1/{{{\mbox{HR}}}}^{[n]}}{\tau }\right),$$

where the notation HR^[*n*]^ denotes the heart rate over the *n*th cardiac cycle, *τ* is the characteristic time for heartrate, and *σ*^[*n*]^ is given by

18 $$\sigma^{[n]} = \left( \mathop{\underbrace{\frac{\alpha_\sigma}{1 + \left(\frac{P_{{\rm{Central}}\,{\rm{Arteries}}}^{[n]}}{P_0}\right)^{\nu}}}}\limits_{{{\rm{Sympathetic}}\,{\rm{Activity}}}} - \mathop{\underbrace{\frac{\beta_\sigma}{1 + \left(\frac{P_{{\rm{Central}}\,{\rm{Arteries}}}^{[n]}}{P_0}\right)^{-\nu}}}}\limits_{{\rm{Parasympathetic}}\,{\rm{Activity}}} + \mathop{\underbrace{\gamma_\sigma}}\limits_{{{\rm{Baseline}}\,{\rm{Activation}}}} \right) \mbox{HR}_0,$$

where *P*_0_ is the baseline Central Arteries pressure, *α*_*σ*_ is the coefficient for sympathetic activity, *β*_*σ*_ is the coefficient for parasympathetic activity, *γ*_*σ*_ is the baseline activation rate, *ν* is the slope parameter, and HR_0_ is the baseline heart rate.

### Reporting summary

Further information on research design is available in the Nature Research Reporting Summary linked to this article.

## Results

### Fixed MAP and CVP

**Fig. 3:**
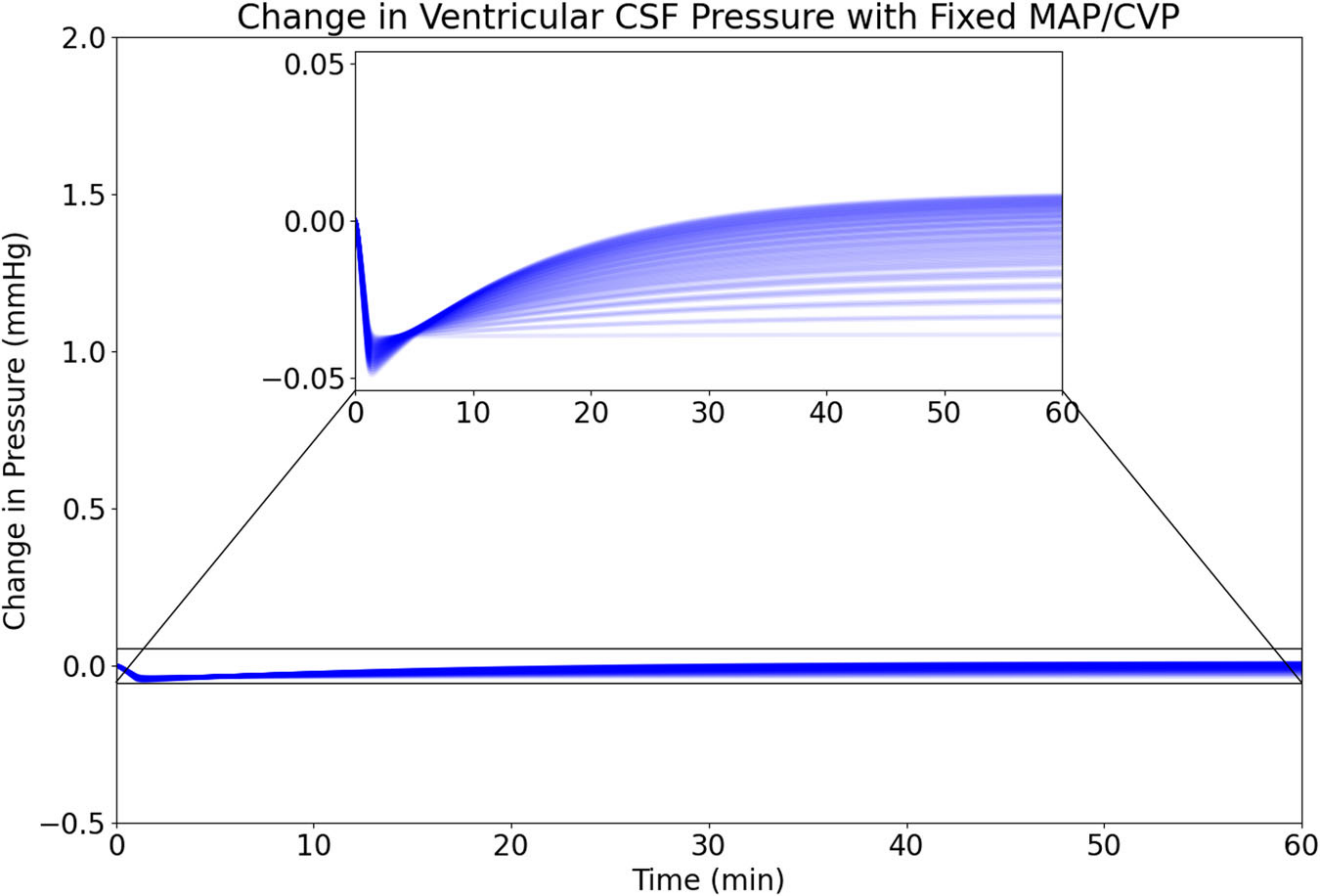
Transient response of ventricular CSF compartment pressure to changes in thoracic compartment pressure when MAP and CVP are fixed. The simulations show <0.05 mmHg change from initial CSF pressure over 91 permutations of MAP and CVP.

The first computational experiment attempts to isolate the direct effect of changes in the pressure of the Thoracic compartment on the Ventricular CSF compartment pressure by holding the MAP and CVP constant while the pressure in the Thoracic compartment varies. Here we investigate 91 combinations of pressures in the Central Arteries and Central Veins compartments consisting of ± 6 mmHg from baseline mean arterial pressure and ±3 mmHg from baseline central venous pressure. As shown in Fig. 3, these 91 combinations exhibit an initial transient in response to the change in pressure in the Thoracic compartment but the Ventricular CSF compartment pressure then relaxes to a steady state with a maximum change of <0.05 mmHg from the initial condition.

### Dynamic MAP and CVP

**Fig. 4:**
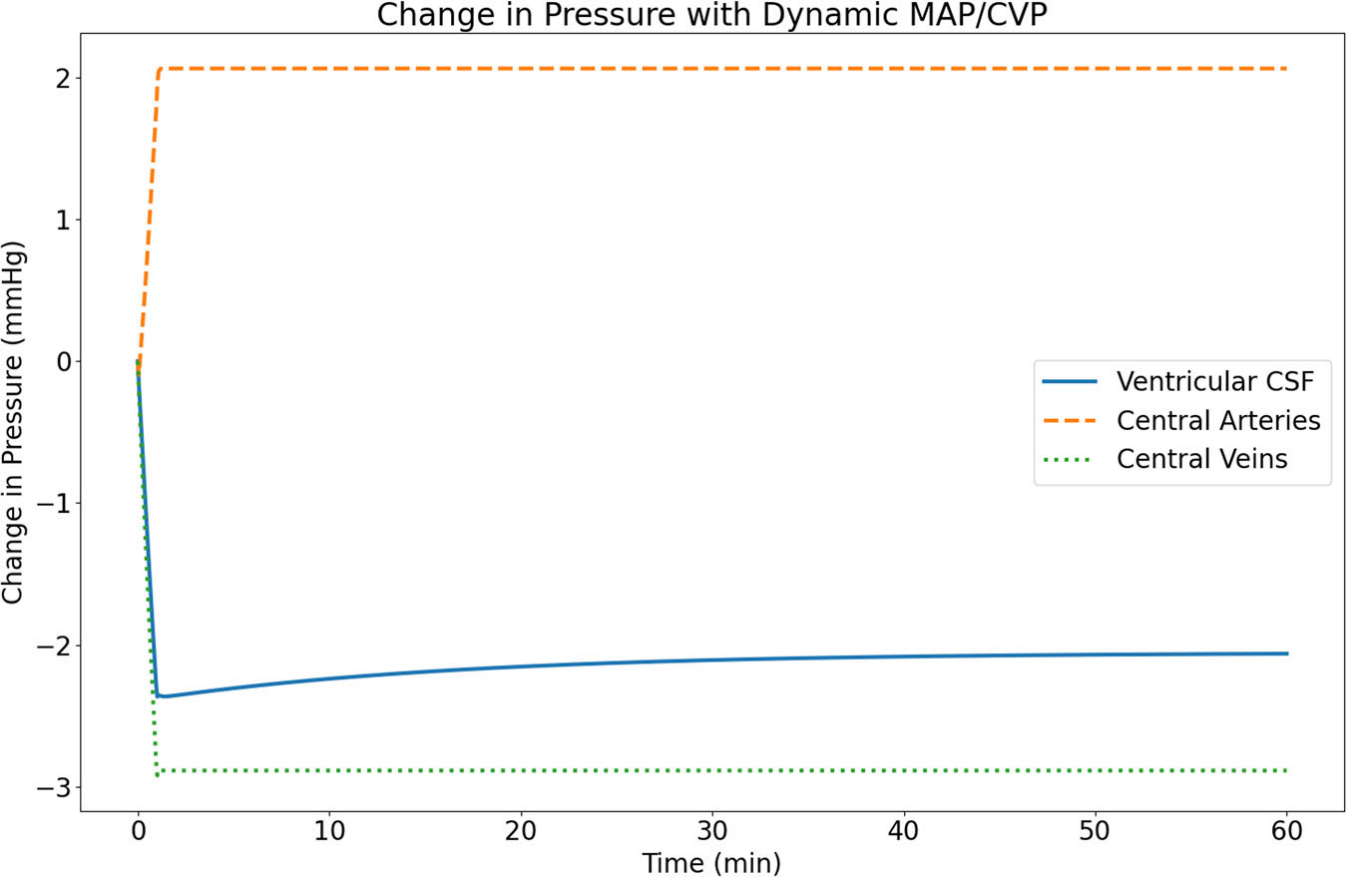
Change in Central Arteries, Central Veins, and Ventricular CSF pressure in response to a drop in Thoracic pressure for dynamic MAP and CVP.

The second experiment allows the MAP and CVP to be driven by a dynamic cardiac output function as described in “Experimental configurations”. This dynamic cardiac output function allows the changes in thoracic pressure to influence the Central Arteries and Central Veins compartments and its direct interaction with the ventricular CSF compartment. As shown in Fig. 4, at the default MAP and CVP values (see Table 3), we observe an ~2 mmHg drop in ventricular CSF pressure in response to the drop in thoracic pressure from -6 mmHg to10 mmHg. Additionally, we observe an ~2 mmHg increase in MAP and a 3 mmHg decrease in CVP. These changes are accompanied by an increase in cardiac output (not shown in the Figure) from 5300 mL min^−1^ to 5750 mL min^−1^.

## Discussion

The presentation of SANS is a complex system response to microgravity and other environmental factors likely linked to cephalad fluid shift in both the cardiovascular and cerebral spinal fluid domains^1^. Although research continues to elucidate the critical pathways associated with vision impairment, the relative contribution of many of these complex fluid shift-influenced features can be well evaluated by computational simulations^35,50^. One such potentially affecting feature is the change of the spinal dura transmural pressure due to the microgravity-induced alterations of thoracic pressure^15^. Establishing the true influence of this relationship through direct measurements of CSF and thoracic pressure remains unfeasible in spacefaring humans leading to computational modeling as one of the most viable means of analysis and insight.

Towards this end, in establishing plausible mechanistic pathways for sustained CSF drainage from cranial ventricular to subarachnoid spaces, this study has explored two computational simulations with a lumped-parameter model—fixed MAP and CVP as well as dynamic MAP and CVP. The Fixed MAP and CVP study seeks to isolate the influence of an acute change in spinal dura transmural pressure from its effects on other physiological parameters. As illustrated in “Fixed MAP and CVP” and Fig. 3, our model does not support the hypothesis that a decrease in thoracic pressure contributes to a substantial or sustained reduction in ventricular CSF pressure resulting from the isolated sequestration of CSF in the spinal CSF spaces (including the spinal dural sac). The changes in pressure attributable to this compliant interaction between the Thoracic compartment and the Spinal CSF compartment are vanishingly small (less than ± 0.5% of the mean value) and recover quickly over a wide range of cerebral perfusion pressures. These findings suggest that the impact of thoracic pressure on ventricular CSF pressure should not be viewed in isolation. Rather, a holistic view of the effects of changes in thoracic pressure and the accompanying CSF drainage and production pathways is necessary.

When we include the impacts of thoracic pressure-induced alterations in transmural pressure on the central arteries, veins, and heart similar to those seen in acute microgravity^21^, we observe a decrease in Ventricular CSF compartment pressure of ~2 mmHg that is consistent with the drop observed in acute microgravity^22,51^. It should be considered that the Dynamic MAP and CVP study (Dynamic MAP and CVP) does not exactly mirror a microgravity environment and the resulting MAP is relatively constant, increasing by 2.1% from the baseline value. This is accompanied by an increase in the cardiac blood flow from 5300 ml min^−1^ to 5750 ml min^−1^. From these findings of the Dynamic MAP and CVP simulation and those from the Fixed MAP and CVP simulation, we infer that the acute microgravity-induced changes in ICP result predominantly from changes imposed on the venous system (Fig. 4) with little or no influence resulting from transmural pressure changes at the spinal dura. Not surprisingly, the Ventricular CSF compartment pressure is observed to mirror CVP changes during the transition phase, further supporting the link of these two pressures in these simulations. Such observations reinforce the importance of investigating the thoracic pressure influence on the cardiovascular system when examining changes in CSF pressure.

Although this study investigates a wide array of parameters and pathways that may influence the movement and sequestration of CSF in the spinal dura, these results should be interpreted in light of the physiological abstractions made in the computational formulation. Specifically, the formulation uses a lumped parameter abstraction of the fluid system, minimal active regulator mechanisms, and a fixed compliance and resistance values related to some compartments. The range of thoracic pressure changes is premised on the approximation of the thoracic pressure changes as being proportional to changes in esophageal pressure observed in microgravity^20^. This implies the range of thoracic pressure changes in our analysis may be wider than those experienced at the spinal dura. In-beat pulsatility of flow is also neglected which ignores potential small alterations in instantaneous transmural pressure at the spine due to phase differences of the compartment pressure waves. However, these limitations would likely have only a slight impact on the short-term model response and would have minimal to no impact on the observations of this analysis.

In conclusion, we have presented a lumped-parameter model of the blood and CSF flow in the head, neck, and thorax. Using this model, we investigated the role of thoracic pressure on CSF pressure and, in particular, the interactions with the Extraventricular CSF space as suggested by Laurie, et al.^15^. The current modeling effort does not support the hypothesis that this direct interaction with the spinal CSF space is sufficient to explain the experimentally observed drop in CSF pressure. However, we do find numerical evidence supporting that the impact of thoracic pressure on the cardiovascular system, in combination with the direct CSF space interaction, is capable of reproducing the observed changes. This work warrants additional investigation in how microgravity can influence the flow dynamics in those areas. In particular, investigating the unloading of tissue weight in microgravity, as suggested by Buckey, et al.^21^, on the compliant Central Veins compartment is future work we intend to explore.

### Supplementary information

Reporting Summary

## Data Availability

Model parameters are from previously published sources (see Tables 1, 2, 3, 4). Model outputs can be obtained with a reasonable request to the NASA-affiliated authors and after appropriate government export control review.

## Code availability

Model source code can be obtained with a reasonable request to the NASA-affiliated authors and after appropriate government export control review.
